# Screening for hepatitis C in psychiatric population

**DOI:** 10.1192/j.eurpsy.2021.267

**Published:** 2021-08-13

**Authors:** F. Icole, J.-P. Bronowicki, C. Jeannoel, P. Besancon, F. Boulanger

**Affiliations:** 1 Pôle De La Déodatie, CHS Ravenel, Mirecourt, France; 2 Gastro-entérologie, CHRU Nancy, Vandoeuvre-lès-nancy, France; 3 Pôle Médico-technique, CHS Ravenel, Mirecourt, France; 4 Pôle Api, CHS Ravenel, Mirecourt, France

**Keywords:** liver, Hepatitis C, comorbidity

## Abstract

**Introduction:**

A meta-analysis from 2016 estimates prevalence of hepatitis C to be superior in people with severe mental illness than general population. In France, positivity for hepatitis C is estimated at 0,75% of general population and 0.3% with a detectable viral load. No recent study was conducted to determine seroprevalence of hepatitis C in population admitted in psychiatric institution.

**Objectives:**

The aims of this study are to determine seroprevalence of hepatitis C in population admitted in psychiatric institution and describe the profile of infected patients.

**Methods:**

From january 2020 to october 2020, screening test for hepatitis C, hepatitis B and HIV was proposed to every patient admitted at the reception unit of Ravenel Hospital. In case of positivity, viral load was realised.

**Results:**

Between January 7^th^ and Octobre 1^st^ , 407 patients greed to the screening test. Among them, 17 (4,2%) were tested positive to hepatits C and viral load was detectable in 9/17 positives, which lead to a 2,2% seroprevalence of hepatitis C infection in the studied population. The patients with positive screening had a mean age of 40 years old. 82% of them were males. 16 admit using intoxicating substances and 10 were still current users at the time of the study. They were hospitalized for addictology purpose (5/17), psychosis (6/17), mood disorder (5/17), personality disorder (2/17), adjustement disorder (2/7). 10/17 had an alcohol use disorder.

**Conclusions:**

This study confirms seroprevalence of hepatitis C infection in psychiatric population is seven times that of general population. This justifies a systematic screening of this population.

**Disclosure:**

No significant relationships.

